# Exposure to Increased Environmental Complexity during Rearing Reduces Fearfulness and Increases Use of Three-Dimensional Space in Laying Hens (*Gallus gallus domesticus*)

**DOI:** 10.3389/fvets.2016.00014

**Published:** 2016-02-29

**Authors:** Margrethe Brantsæter, Janicke Nordgreen, T. Bas Rodenburg, Fernanda M. Tahamtani, Anastasija Popova, Andrew M. Janczak

**Affiliations:** ^1^Animal Welfare Research Group, Department of Production Animal Clinical Science, NMBU School of Veterinary Science, Oslo, Norway; ^2^Behavioural Ecology Group, Wageningen University, Wageningen, Netherlands

**Keywords:** laying hen, chicken, fearfulness, fear, stress, rearing, behavior

## Abstract

The complexity of the rearing environment is important for behavioral development and fearfulness. The aim of this study was to test the hypothesis that laying hens reared in a complex aviary system with exposure to mild intermittent stressors would be less fearful, less sensitive to stress, and would use elevated areas of the pen more often as adults than hens reared in a barren cage environment. Laying hens (*N* = 160) were housed in the same rearing house; half of the birds (*n* = 80) in an aviary and the other half (*n* = 80) in cages. At 16 weeks of age, the birds were transported to the experimental facilities. Their behavior was recorded at 19 and 23 weeks of age and analyzed by analysis of variance on individual scores for a fearfulness-related principal component generated using principal component analysis. The results indicate that aviary-reared birds have lower levels of fearfulness compared with cage-reared birds both at 19 weeks and at 23 weeks of age. When comparing the response induced by initial exposure to a novel object at 19 and 23 weeks of age, more aviary-reared birds tended to fly up at 19 weeks compared to the cage-reared birds, indicating a tendency toward a more active behavioral response in the aviary-reared birds than in cage-reared birds. There was no difference between treatments in the flight response at 23 weeks. The groups did not differ in defecation frequency or the concentration of fecal corticosterone metabolites at either age. At 19 weeks, observation of the spatial distribution in the home pens indicated that more aviary-reared birds spent time on the low perch, the elevated platform, and the upper perch, compared to the cage-reared birds. However, at 23 weeks of age, these differences were no longer detected. The results of this study support the hypothesis that increased environmental complexity during rearing reduces fearfulness of adult laying hens.

## Introduction

Fear normally functions to protect an animal from danger (1). However, exposure to fear-inducing stimuli is also a potent stressor associated with activation of the hypothalamic–­pituitary–adrenocortical (HPA) axis. Therefore, fear may have negative consequences for animal welfare and productivity if the fear response is exaggerated, inappropriate or expressed in a restrictive environment (2–5). Fearfulness is the predisposition of an individual to be easily frightened (1, 6) and is influenced both by genetic and developmental factors.

The early environment may have a large impact on the development of fearfulness and associated activation of the HPA axis in response to stressors (6–9). Exposure to increased environmental complexity during rearing has been found to reduce fearfulness during adulthood in several species including mice (10), pigs (11), and chickens (12). For laying hens, the housing system during rearing is a major source of environmental variability, illustrated by the large difference between cage- and aviary-rearing systems, but few studies have tested for effects of the rearing system on later fearfulness in laying hens. Johnsen et al. (12) compared floor-housed adult birds reared on sand, straw, or wire from 0 to 4 weeks and found that birds reared on wire were most fearful as indicated by longer durations of tonic immobility in response to manual restraint. Anderson and Adams (3) compared cage-housed adult birds reared in a floor or cage system and found that floor-reared birds were more active and displayed more flighty responses to a human than cage-reared birds. A similar study failed to find differences in escape or tonic immobility responses between floor- or cage-reared laying hens housed in cages as adults (13). Other studies testing for effects of exposure to varying degrees of environmental complexity confound effects of rearing and housing of adult birds (14, 15). To the best of our knowledge, there are no previous studies comparing the effects of rearing in a complex aviary system with rearing in a barren cage environment on fear responses in birds housed in the same environment as adults. This knowledge is required for a better understanding of the characteristics of laying hens reared in aviaries or cages under conventional production conditions.

There is a consensus that an individual’s fearfulness can be quantified by observing its response to potentially dangerous animate or inanimate objects (6, 16–18). Novel object (NO) tests and human approach tests measure the conflicting motivations to approach and avoid an object as described by Miller’s Model (19, 20). According to Miller’s Model, an animal will approach an aversive object up to the point at which the motivation to avoid the stimuli becomes as strong as the motivation to approach it (19–22). Fearful animals exposed to potentially dangerous objects typically show escape attempts, avoidance, longer latencies to approach and immobility as well as elevated activation of the HPA axis or sympathetic nervous system, depending on contextual variables and the animals’ behavioral strategy (17, 23). However, sometimes it is unclear which variables represent the best measures of fearfulness in a given test situation.

Previous studies also indicate that early experience with a more complex environment may increase the ability of birds to use elevated perches and improve their ability to solve spatial tasks as adults (24–26). This is likely due to effects of sensory stimulation, locomotor experience, and exercise of brain structures underlying cognitive processes as well as neuromuscular systems (26). On this basis, one would expect birds reared in a complex aviary system to use elevated areas of the home cage more often than birds reared in barren cages.

The aim of this study was to test the hypothesis that birds reared in a complex aviary system with exposure to mild intermittent stressors would be less fearful, less sensitive to stress, and use elevated areas of the pen more often as adults than laying hens reared in a simpler cage environment.

## Materials and Methods

### Subjects and Rearing Treatments

The study was conducted using non-beak trimmed, female Dekalb white chickens (*Gallus gallus domesticus*), aged 0–23 weeks with normal health status. Birds were hatched at a commercial hatchery and then reared in separate corridors in a single room until 16 weeks of age. Each corridor in the room contained either a cage- or an aviary-rearing system. The housing system in the single room in which all birds were housed was Natura Primus 1600 (Big Dutchman; http://www.bigdutchmanusa.com) designed for aviary-rearing of laying hen pullets. This system consists of cages stacked in three tiers placed on either side of a corridor for allowing inspection by the caretaker. Cage dimensions are 120 cm × 80 cm × 60 cm (length × width × height). Each aviary cage contains a 120 cm feed trough, one 120 cm perch, and five drinking nipples. All cages can be opened at the front, so that birds can move between each tier and the floor of the corridor. Ramps run from the floor to the second tier to increase ease of access for pullets. When cage doors are in the open position, perches extend from the front of the first and second tiers. The density was 25 birds/m^2^ for both treatments during the first 4 weeks of life.

At delivery to the rearing farm immediately following hatching, all chicks were initially placed in cages on the first and second tiers. Chick paper covered 30% of the wire mesh floor of the cages in sufficient amounts to last until the birds were released out in the corridors. At 4 weeks of age, aviary-reared birds (half of the birds in the house) were released from these cages by opening cage doors and allowed to move between the floor of the corridor and each aviary tier on each side of the corridor until the end of the rearing phase at 16 weeks of age. Aviary-reared birds and cage-reared birds were housed in separate corridors throughout the rearing phase. The cage-reared birds (the other half of the birds in the house) were kept inside cages of the first and second tiers until the end of the rearing phase at 16 weeks of age, after which a random subset of birds reared according to each treatment was moved to the experimental facilities.

During rearing, all birds were exposed to the same light intensity, light schedule, and temperatures, as recommended by the General Management Guide for Dekalb White Commercial Layer (27). They were provided with *ad libitum* access to feed using a chain dispersal system and *ad libitum* access to water. The feed type was conventional pullet feed produced and sold by Felleskjøpet, Norway (“Kromat oppdrett 1” for 0- to 6-week-old birds, “Kromat avl egg 1” for 6- to 8-week-old birds, and “Kromat oppdrett 2” for 8- to 15-week-old birds).

### Housing, Feeding, and Lighting at Experimental Facilities

The house was 60 m × 20 m and contained 52,000 chickens in total. At 16 weeks of age, 240 birds from each rearing system (480 birds in total) were transported 490 km by car in transport crates to the experimental poultry facilities at the Norwegian University of Life Sciences, Campus Ås, Norway. At the experimental facilities, they were housed in custom built pens in two adjacent rooms. The two rooms were identical in size and shape and measured 5.90 m × 4.90 m. Each room contained 22 pens. Twenty pens per room contained experimental birds and the remaining two contained reserve birds that were not used in the study. Each room thus contained a total of 240 experimental birds. Each pen’s dimensions were 120 cm × 80 cm × 200 cm (length × width × height), and pens were built out of wire mesh on a wooden frame. Each pen contained a wooden nest box (40 cm × 60 cm × 20 cm), an elevated platform (80 cm × 50 cm) at a height of 110 cm, and two perches (80 cm long), one at 70 cm and one at 140 cm above the floor. Each pen contained 12 birds. Birds were housed in mixed groups of six aviary-reared birds and six cage-reared birds per pen (see the Discussion section for a discussion of pros and cons of mixed housing). The experimental pens were numbered 1–20 (room 1) and 21–40 (room 2). On arrival, the birds from both treatments were randomly assigned to a pen. All the birds were fitted with a transparent thin plastic band around the right leg. The end of the plastic band was cut off at 90° (cage-reared birds) or at 45° (aviary-reared birds) to identify the treatment group to which each bird belonged. Also, colored spray paint was used to ease the identification of each treatment group from a distance and thus minimize the handling necessary to collect birds before testing. The birds were sprayed with blue spray paint from wing to wing or with dark green paint from the shoulder blades to the tail. Both markings were allocated to both treatment groups (alternating between pens) to preclude confounding effects of treatment and type of color marking. This identification system was used to ensure that observers were blind to treatment conditions when scoring the distribution of birds in the home pen.

The experimental facility in which adult hens were housed operated on a light cycle that was altered according to recommendations by the Dekalb Management Guide (27). This involved exposure to 100 lux for 24 h after arrival followed by 5–7 lux during the light cycle. Feed was provided *ad libitum* using a circular feeder (50 cm in diameter) hanging 20 cm above ground level. Water was provided *ad libitum* by nipple drinkers (two per pen) mounted 30 cm above ground at the back of the cage. Birds were manually fed with Fjør Oppdrett Lett (Felleskjøpet) until start of lay (16- to 18-week-old birds) and Fjør Egg (Felleskjøpet) until the end of the experiment (24-week-old birds).

### Behavioral Tests in the Test Arena

The behavioral tests were performed at 19 weeks (*n* = 80) and 23 weeks of age (*n* = 80). Each bird was only tested once. All birds were tested in a combined voluntary human approach and a NO test. During the test periods, two birds from 10 different pens (five pens per room) were tested each day over a four-day period. From each pen, two aviary-reared birds and two cage-reared birds were tested. The test order of birds was balanced across the room, the distance from pen to the door, and the two rearing treatments. When entering a pen to test a bird, a bird was pseudo-randomly chosen from the floor, the perches, or the elevated platform by the handler. At the first time of testing (19 weeks of age), all birds came from pens with odd numbers. At the second time of testing (23 weeks of age), all birds came from pens with even numbers. The procedure was otherwise the same as for testing at 19 weeks.

**Figure 1:**
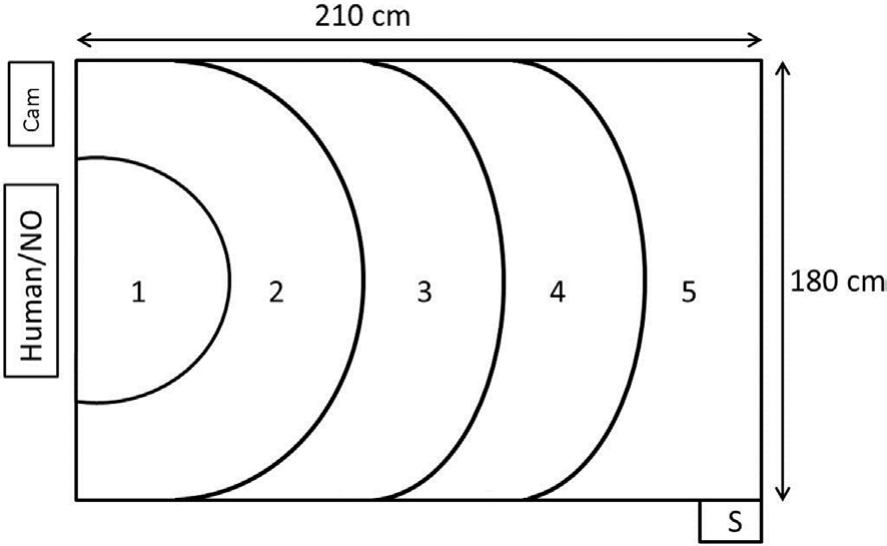
**Figure illustrating the test arena where the human approach test and the novel object test was performed**. The human or novel object (NO) was positioned just outside the transparent wall. The birds were placed in the arena through the start box (S). The EthoVision XT camera was mounted in the ceiling above zone 3 and pointed down. There was an additional camera (cam) next to the human/novel object. Numbers 1–5 represent zones of increasing distance from the test stimuli.

The test room measuring 4.90 m × 5.90 m contained a test arena measuring 210 cm × 180 cm × 120 cm (length × width × height) in one corner. Three of the walls were black and opaque, whereas the fourth wall consisted of netting and was, therefore, transparent. The human or the NO was positioned 20 cm outside the netting. When sitting in front of the arena, the stimulus person in the human approach test looked directly toward the arena. The light intensity (measured at chicken height in the test arena) was 7 lux and the sound level between 40 and 60 dB (depending on fan speed). Every morning of the test days, reserve birds from the extra pens that were not used in the experiment were picked up to standardize disturbance of birds before testing. In this way, also the test animals that were tested first had already experienced birds being caught and handled in a different pen prior to testing. The test animals were individually carried on the arm of the worker a distance of 10–20 m from the home pen to the test arena. The time from approaching the bird in the home pen to entering the test arena was 54.7 s (mean) ± 8.24 (SD). The recording of the birds was done by two cameras: one (Panasonic, WV-CP500/G) was suspended from the ceiling, positioned so that it faced the middle of the test arena and connected to a computer with EthoVision XT 10 (Noldus Information Technology, Wageningen, The Netherlands), and the other camera was a camcorder (Canon, Legria HFM56) mounted on a tripod that recorded birds from a position adjacent to the human or NO (Figure 1).

A single observer functioned as the stimulus person in all the human approach tests. She was positioned 20 cm outside the netting wall, wore black tights and a blue overall. She sat quietly facing the arena, avoided eye contact with the bird, and tried to keep movements to a minimum during testing. The bird was put into a start box outside one corner of the test arena farthest away from the human, so that it entered into zone 5 (Figure 1). The bird was placed into the arena by another experimenter so that by the time the bird entered the arena, the stimulus person was already positioned on the chair. The duration of the test was 5 min. After testing, the bird was left in the arena and exposed to the NO test, as described below.

The stimulus object for scoring the flight response for the NO test was a beige umbrella. The flight response was scored as the umbrella was opened at the beginning of the NO test. As soon as the human approach test was completed, the stimulus person had 5 s to open the umbrella, place it on the chair in front of the arena, and move out of sight of the bird (see Figure 1). The duration of the NO test was 5 min. Birds were returned to the home pen directly after the testing.

### Behavioral Registrations in the Human Approach and Novel Object Tests

EthoVision XT (Noldus Information Technology, Wageningen, The Netherlands) was the software used to calculate the following variables: distance moved during each test, duration of time spent standing still, and the total time spent in areas of the arena closest to the stimuli (zones 1–4; see Figure 1). The birds’ response when the umbrella was opened at the beginning of the NO test was categorized as flight or no flight by a blind observer.

### Collection and Analysis of Fecal Samples

After the NO test had been completed, the animal was marked with an additional thin, yellow plastic leg-ring to make sure that the same animal would not be tested again. The number of droppings during the 10 min (defecation frequency) in the test arena was recorded, and all feces were collected for analysis of corticosterone metabolites. The fecal samples were stored in a freezer at −80°C until analysis using an enzyme immunoassay (EIA). Droppings were extracted with 60% methanol [0.5 g + 5 ml; (28)] and corticosterone metabolites were measured in an aliquot (after 1:10 dilution in assay buffer) of the supernatant. Measurement was performed with an EIA, which has been successfully validated for non-invasive evaluation of adrenocortical activity in chicken [for details of the assay, see Ref. (29)]. Extraction was performed at NMBU. The EIA was performed at the University of Veterinary Medicine, Vienna.

### Behavioral Registrations in the Home Pen

At 19 and 23 weeks of age, the spatial distribution of birds in the home pen was recorded. Observations were done twice daily between 09:30–10:00 and 15:00–16:00 by two observers balanced across the two housing rooms. The observer walked down the aisle of the room, counting the number of birds with each type of spray mark that were (a) perching on the upper perch, (b) sitting on the elevated platform, (c) perching on the low perch, or (d) on the floor. The number of birds positioned on the floor was calculated by subtracting the birds that were counted from the total number of birds in the pen. The recording took 10–15 min per room.

### Ethical Statement

This study was approved by the Institutional Animal Care and Use Committee at the Norwegian University of Life Sciences under ID number 6190.

### Data Treatment and Statistical Analysis

The statistical software JMP version 11.0 was used for all statistical analysis (SAS Institute Inc., NC, USA) except where stated otherwise. The pattern of correlation between the continuous test variables (distance moved, duration of standing, and duration of time spent in the four zones closest to the stimuli) in the behavioral tests was analyzed using principal component analysis (PCA) in order to interpret and reduce the number of variables. The variables included in the PCA were total distance moved in the duration of the test, duration of standing still, and the duration of time spent close to the human or NO for both tests, so that the PCA was run on six variables in total. The distribution of variables indicated that no transformations were necessary prior to running PCA. A detailed description of the PCA is provided in Hatcher (30). Principal components were retained for further interpretation if they had an Eigenvalue >1 (the Kaiser criterion), and the scree plot showed a clear separation between retained and unretained principal components and they were interpretable (30). Furthermore, variables were required to have a loading of >0.40 (30). In accordance with a study by Campler et al. (31), no rotation was used. Rotation was not used partly because only one principal component was retained (30), meaning that rotation would be meaningless, and because we were interested in the empirical relationships between variables related to general fearfulness and not in separating these into different stimulus-specific dimensions. The component that was retained was used to generate component scores for individual birds in order to test for treatment effects using ANOVA.

Principal component scores were checked to confirm that they fulfilled the assumptions of general linear models (independence, normality of residuals, homogeneity of variance, and linearity). The ANOVA model was *Y* = pen’ + treatment + pen’ × treatment. Because two hens from the same treatment were tested from each pen, we used pen and not hen as the experimental unit to avoid pseudo replication. Pen was a random factor and treatment was a fixed factor.

The flight response when birds were first exposed to the NO was categorized as a nominal variable (flight or no flight). The effect of treatment on whether birds showed a flight response was analyzed using logistic regression in Stata (STATA SE 14.0 for Windows). Analysis was run separately for the two ages (19 and 23 weeks of age). Both treatment (aviary vs. cage) and the zone (1–5) in which the bird was positioned when the umbrella opened were included in the model. For treatment, aviary-reared birds were compared to cage-reared birds, and for zone, zones 1, 2, 3, and 4 were separately compared with zone 5 (start zone farthest from the NO). The interaction between treatment and position was tested in the model but was not significant and led to a higher Akaike information criterion and Bayesian information criterion and was therefore removed. Very few birds were positioned in zone 1 when exposed to the NO: two aviary-reared birds at 19 weeks of age, both showing a flight response, and one aviary-reared and one cage-reared bird at 23 weeks of age, none of them showing a flight response. Thus, we did not have all combinations of treatment and flight response for zone 1 at any age, and the comparison between birds starting in zones 1 and 5 could not be carried out. In consequence, the four observations from zone 1 were removed from the dataset, giving a total of 78 data points per age. Odds ratios (OR) and *p*-values are reported. The significance of the whole model was assessed by the likelihood ratio test.

Flight in response to sudden stimulation is sometimes used as an indicator of fearfulness. The relationship between individual scores for the principal component related to fearfulness and the flight response, when exposed to the umbrella, was therefore tested by logistic regression (STATA SE 14.0 for Windows). Flight response was treated as a dependent variable, and the principal component score was used as an independent covariate.

The defecation frequency was analyzed using Fisher’s exact test. A total of 148 fecal samples were obtained for analysis of corticosterone metabolites. The corticosterone metabolite data fulfilled the assumptions of General Linear Models and were analyzed using the model described for the principal component scores. The results of the defecation frequency are presented as median, 25th and 75th percentiles, whereas the results for the concentration of corticosterone metabolites are presented as means ± SDs. The data from the home pen observations were treated as follows. The number of hens from each treatment on the top perch, the elevated platform, and the low perch was counted and then divided by the total number of hens from the relevant treatment to give the percentage of birds from each treatment that were found on each of the three different levels. We then calculated the average number of birds over the four days in each position in the pen during the periods of observation. The resulting data were analyzed by Wilcoxon matched-pairs signed-rank sum test, while treating the relative number of aviary-reared birds and the relative number of cage-reared birds in each position as matched pairs. The median, 25th and 75th percentiles, are given for the different locations.

## Results

Between the time of delivery at the experimental facilities and the time of study, three animals were excluded due to injuries including two cage-reared birds in two different pens and one aviary-reared bird in a third pen.

### Principal Component Analysis and Analysis of Principal Component Score

**Figure 2:**
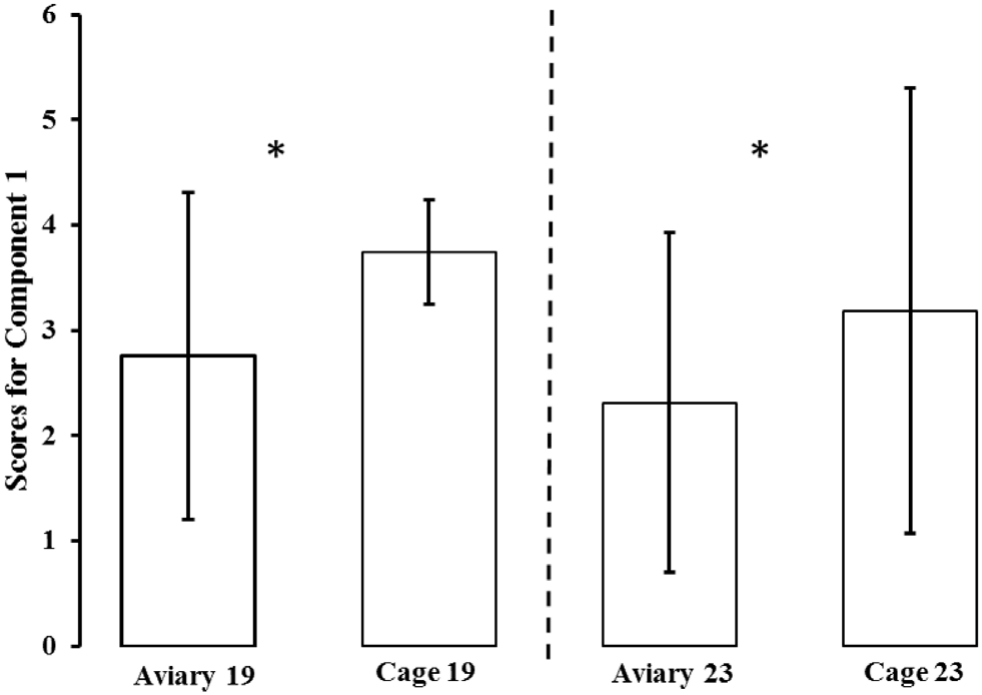
**Mean ± SD scores for Component 1 for aviary-reared and cage-reared birds at 19 and 23 weeks of age**. Principal component analysis was conducted to generate individual scores for a component measuring “fearfulness” (scores for Component 1). To avoid negative values, three was added to all component scores in the figure. Significant differences are marked *.

The PCA generated six components (see Table 1). Only the first component (Component 1) fulfilled the criteria for interpretation (based on Kaiser criterion and scree plot). Component 1 accounted for 55% of the total variation in the data and was highly correlated to all of the test variables. Except for duration of standing still in both tests, all loadings on Component 1 were negative. A high score on Component 1 indicated that a bird spent more time in the area farthest from the NO and human, indicating a high degree of avoidance or a lack of approach. A high score also indicated that a bird spent less time moving and more time standing still. The aviary-reared birds had a lower score for Component 1 compared with the cage-reared birds both at 19 weeks [aviary-reared: −0.2439 ± 1.5560. cage-reared: 0.7437 ± 1.7232. *F*_(1,19)_ = 5.6609; *p* = 0.0280] and at 23 weeks of age [aviary-reared: −0.6864 ± 1.6178. cage-reared: 0.1865 ± 2.1125. *F*_(1,19)_ = 4.4907; *p* = 0.0493; Figure 2].

**Table 1:** **Loading matrix from the principal component analysis (PCA) based on behavioral tests at 19 and 23 weeks of age**.

	Comp. 1	Comp. 2	Comp. 3	Comp. 4	Comp. 5	Comp. 6
**Human approach test**						
Distance moved (cm)	**−0.8908**	−0.2171	0.2030	−0.0276	−0.2885	−0.1846
Duration standing still (s)	**0.7819**	0.2413	−0.3455	**−0.4371**	−0.0718	−0.1217
Duration 1–4 (s)	**−0.6396**	**−0.6356**	−0.1972	−0.3168	0.2164	0.0291
**Novel object test**						
Distance moved (cm)	−**0.8421**	0.3959	−0.0666	−0.2441	−0.1937	0.1808
Duration standing still (s)	**0.7425**	**−0.5504**	−0.1903	0.0536	−0.3079	0.1088
Duration 1–4 (s)	**−0.5007**	0.0931	**−0.8190**	0.2612	−0.0060	−0.0414
**Eigenvalue**	3.325	0.9778	0.9108	0.4229	0.2676	0.0960
Variation explained (%)	55.417	16.296	15.179	7.048	4.460	1.600
Cumulative variation (%)	55.417	71.714	86.893	93.940	98.400	100.00

*The PCA generated six components (Comp. 1–6). Component loadings >0.40 are written in bold. Duration 1–4 indicates the duration of time spent outside of the area farthest away from the novel object or human*.

### Flight Response

**Figure 3:**
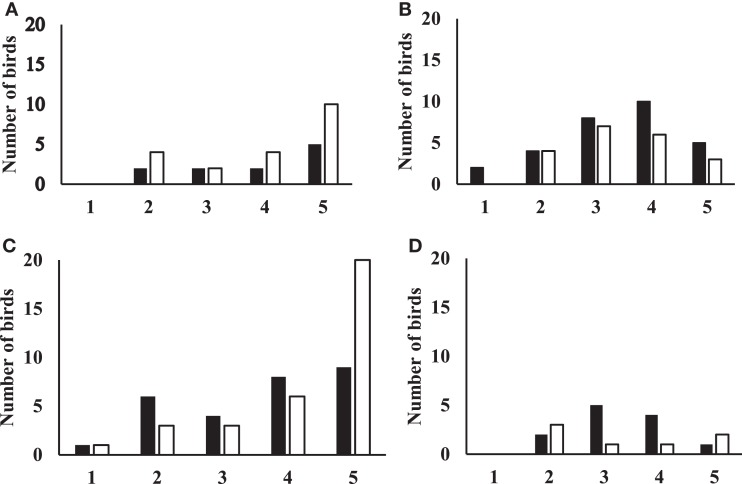
**Figures showing the flight response for aviary-reared and cage-reared birds at 19 and 23 weeks of age**. Black columns indicate aviary-reared birds. White columns indicate cage-reared birds. The *x*-axis indicates the zone. Zone 1 was closest to novel object and zone 5 was the start zone farthest away from the novel object. The *y*-axis indicates the number of birds that did not fly at 19 weeks of age **(A)**, the number of birds that flew at 19 weeks of age **(B)**, the number of birds that did not fly at 23 weeks of age **(C)** and the number of birds that flew at 23 weeks of age **(D)**.

Flight responses are shown in Figure 3. For data from birds tested at 19 weeks of age, the model was highly significant (Likelihood ratio test: chi-square = 13.68, *p* = 0.0084). Aviary-reared birds tended to have higher odds of showing a flight response than cage-reared birds (OR = 2.4, *p* = 0.086). Birds in zones 3 and 4 had higher odds of showing a flight response compared to birds in zone 5 (zone 3: OR = 7.1, *p* = 0.007; zone 4: OR = 4.9, *p* = 0.017). There was no interaction between treatment and zone.

For birds tested at 23 weeks, the model tended to be significant (Likelihood ratio test: chi-square = 8.88, *p* = 0.064). At 23 weeks of age, there was no difference between aviary-reared and cage-reared birds in the probability of showing a flight response (OR for aviary-reared compared to cage-reared birds: 1.4; *p* = 0.6). However, birds in zones 2 and 3 had higher odds of showing a flight response than birds in zone 5 (zone 2: OR = 5.0, *p* = 0.055; zone 3: OR = 7.4, *p* = 0.018). There was no interaction between treatment and zone.

The OR of flight vs. no flight was not significantly influenced by the principal component score (OR = 0.89 ± 0.08; *z* = −1.34; *p* = 0.18).

### Defecation Frequency and Corticosterone Metabolites in Feces

For both treatments in both weeks, the defecation frequencies were low (overall median = 1; 25th–75th percentile = 1–2), and no significant effects of treatment or week were found (Fisher’s exact test; *p* > 0.570). There was no effect of treatment on the concentration of corticosterone metabolites either at 19 weeks (mean ± SD) [aviary-reared birds: 174 ± 45 ng/g; cage-reared birds = 183 ± 60 ng/g; *F*_(1,18.63)_ = 0.4728; *p* = 0.5002] or at 23 weeks of age [aviary-reared birds: 151 ± 67 ng/g; cage-reared birds: 166 ± 70 ng/g; *F*_(1,16.21)_ = 1.1421; *p* = 0.3009].

### Home Pen Data

At 19 weeks, the aviary-reared birds were observed significantly more often on the top perch, elevated platform, and low perch, compared to the cage-reared birds (Table 2). At 23 weeks of age, there was no longer an effect of treatment on the distribution in the home pen.

**Table 2:** **Showing results of the Wilcoxon test for distribution of birds in the home pens at 19 and 23 weeks of age**.

		Aviary-reared birds		Cage-reared birds		Test statistic *S*	***p***-value
		Median	25th–75th percentile	Median	25th–75th percentile
19 weeks	Top perch	16.67	10.42–22.92	5.21	2.08–14.06	−289	<0.0001
	Elevated platform	19.20	13.02–25	12.08	6.25–22.08	−183	0.0088
	Low perch	20.83	14.36–22.92	12.5	10.42–18.44	−204.5	0.0045
23 weeks	Top perch	9.38	4.69–14.58	6.25	2.08–10.42	−41	0.1014
	Elevated platform	12.5	7.71–22.40	6.25	4.17–16.15	−34.5	0.1381
	Low perch	15.63	10.94–18.75	13.54	8.33–18.75	−30	0.2364

*At 19 weeks, aviary-reared birds were positioned significantly more often on all elevated areas in the home pen, whereas at 23 weeks of age there was no difference between the treatment groups*.

## Discussion

### Summary

The aim of this study was to test the hypothesis that exposure to increased environmental complexity during rearing reduces fearfulness and increases use of three-dimensional space in adult laying hens. The PCA identified one meaningful component that was used to generate individual scores related to fearfulness, as discussed below. Analysis of treatment effects on scores for this component confirmed that aviary-reared birds housed in the more complex environment were less fearful than cage-reared birds both at 19 and 23 weeks of age. There was a tendency for more aviary-reared birds to fly when startled compared to cage-reared birds at 19, but not at 23 weeks, suggesting that birds reared in the more complex environment initially tend to have a more active behavioral response to the acute fear-inducing stimuli. Observation of the birds’ behavior in their home pens indicated a transitory effect of rearing in which aviary-reared birds more often used the elevated parts of the pen than cage-reared birds at 19, but not at 23 weeks of age, suggesting that rearing in a more complex environment increases three-dimensional spatial orientation or motor skills. The rearing treatment had no effect on defecation frequency during behavioral testing or on the concentration of fecal corticosterone metabolites at either age. The latter finding suggests that there were no treatment effects on basal HPA-axis activity.

### Principal Component Analysis

Fearfulness is the predisposition to avoid different potentially dangerous stimuli as measured using the duration of time spent farthest away from the NO or human in our behavioral tests. Therefore, we interpret Component 1 as reflecting fearfulness. Other variables loading on Component 1 indicated that more fearful birds moved less and spent more time standing still. This corresponds well to interpretations of behavioral inhibition and lack of locomotion as a frequently used indicator of fearfulness in laying hens (17). Our interpretation also corresponds well with a similar study indicating that standing or sitting alert and locomotion recorded in some fear-inducing situations in laying hens were related to the same principal component (31). The remaining components in the current study were related to such few variables that interpretation would be highly speculative.

### Treatment Effects on Fearfulness

Analysis of treatment effects on scores for Component 1 interpreted as fearfulness as discussed above, confirmed that aviary-reared birds housed in the more complex environment were less fearful than cage-reared birds at both ages. This corresponds well to findings by Brantsæter et al. (32) in which cage-reared birds were more hesitant than aviary-reared birds to approach a NO in their home cage. The current study used PCA analysis to generate a fearfulness score that took account of six variables across two different test conditions in which birds were exposed to a variety of stimuli. The PCA score used in the present study may be a better measure of fearfulness than the single response variable used by Brantsæter et al. (32), as the latter may be more stimulus specific.

### Treatment Effects on Flight Response

Aviary-reared birds tended to fly more when the umbrella was opened than the cage-reared birds at 19 weeks, but not at 23 weeks of age. The flight response provides information about how actively the birds responded when exposed to unexpected, abrupt event. The flight response is similar to responses observed in flocks of birds living under production conditions that respond to sudden exposure to novel stimuli. Such panic responses may result in clumping and mortality by suffocation of birds located at the bottom of heaps that might form. Therefore, the tendency for aviary-reared birds to be more predisposed to fly in response to sudden exposure to novelty suggests that they might have more trouble with clumping in loose housing systems than cage-reared birds. However, this disadvantage must be weighed against the many disadvantages of housing cage-reared birds in aviaries regarding problems with navigation (26) and use of perches and nest boxes (24, 25). Some authors interpret flight responses as an indication of elevated fearfulness (3, 33). However, this interpretation is questionable in light of the findings in the current study showing a lack of any relationship between fearfulness as indicated by principal component scores and flight response. We propose that flight in response to acute exposure to novel stimuli in laying hens rather reflects the coping style of birds. This interpretation would suggest that rearing in a more complex and challenging environment tends to make birds more proactive (34).

An aspect of our experimental design that may have influenced the rearing effect on flight is the position of the bird in the arena at the time the umbrella was opened. Aviary-reared birds, which came closer to the human during the voluntary human approach test, might have been more intensely stimulated than birds that were positioned further away. If this is correct, it means that aviary-reared birds would have been exposed to a higher degree of stimulation when the umbrella was opened. This may have increased the likelihood of flying in this treatment group. However, the lack of interaction between treatment and zone indicated that this was not the case.

### Defecation and Corticosterone Metabolites

To the authors’ knowledge, no previous studies have compared defecation frequency in birds subjected to rearing conditions with different degrees of environmental complexity. In rodents, defecation frequency is widely used to assess the stress levels experienced by the animal in behavioral tests (1, 35–38). In chickens, defecation frequency is not as common to measure but is sometimes reported as a measure of underlying fearfulness (39–42). The present study did not detect an effect of environmental complexity during rearing on defecation frequency. The concentration of corticosterone metabolites in the feces is considered as an indirect measure of the level of circulating plasma corticosterone. Measuring fecal corticosterone metabolites is an increasingly used method for non-invasive quantification of chronic stress (43–45). Plasma corticosterone is mainly metabolized by the liver and can be found in the feces approximately 4 h after an induced increase in blood levels (46). Therefore, a treatment effect on corticosterone metabolite excretion would indicate higher basal activity in the HPA axis, but this was not found in the current study. Future studies could include tests for rearing effects on the HPA response to acute stress using analysis of blood samples.

### Space Use in the Home Pen

At 19 weeks of age, most of the cage-reared birds were observed on the floor of the home pens, whereas more aviary-reared birds were positioned on the perches or the elevated platform. At 23 weeks of age, this treatment effect had disappeared. The effect at 19 weeks of age suggests that aviary-reared birds are more aware of perches and elevated areas of the pen or that they have better-developed motor systems. This finding and interpretation corresponds to previous studies (24–26). This temporal development in treatment effects on the use of elevated areas of the home pens corresponds well to the treatment effects on the flight response as previously discussed.

### Pros and Cons of Housing Both Treatments in the Same Pens

In this study, we cohoused birds from both treatments. This was considered necessary to increase power and exclude the possibility of confounding effects of pen and treatment. However, fearful individuals can influence their conspecifics (47, 48). At the most extreme, this transmission can cause whole flocks to panic (2). By housing the treatment groups in mixed pens, the birds could influence each other and become more similar over time. In the present context, this would be a conservative source of error, tending to reduce the likelihood of finding treatment effects.

### Animal Welfare Implications

The current study was conducted when the birds were between 19 and 23 weeks of age. At 19 weeks of age, birds have been transported from the rearing farm and are starting to lay. This is therefore a time in the life of laying hens at which their ability to cope with fear-inducing environmental changes and challenges may be especially important for their welfare and productivity. The present findings, therefore, suggest that laying hens reared in a more complex system are better equipped to cope with the challenges to which they are exposed to around the onset of lay.

## Conclusion

This study confirmed our hypothesis that environmental complexity during rearing has an effect on the development of fearfulness in laying hens. The fear tests conducted at 19 and 23 weeks of age revealed that aviary-reared birds were less fearful compared to the cage-reared birds. The rearing treatment did not affect defecation frequency during testing or basal corticosterone metabolite concentrations. The latter suggests that varying environmental complexity does not influence basal activity in the HPA axis.

## Author Contributions

MB collected the data, analyzed the data, did practical work at the study site, and wrote the first draft of the article; JN did practical work at the study site and collected and analyzed the data; AP did practical work at the study site and collected data; TR contributed to conception of the study; AJ led the project, participated in conception and design of the study, and did practical work at the study site. All authors contributed to planning the study, writing the manuscript, and approved the final version.
